# Estimation of Excited-State Geometries of Benzene and Fluorobenzene through Vibronic Analyses of Absorption Spectra

**DOI:** 10.1021/acsomega.2c04615

**Published:** 2022-09-01

**Authors:** Muhammet Erkan Köse

**Affiliations:** Department of Chemistry, Kocaeli University, Izmit, Kocaeli 41001, Turkey

## Abstract

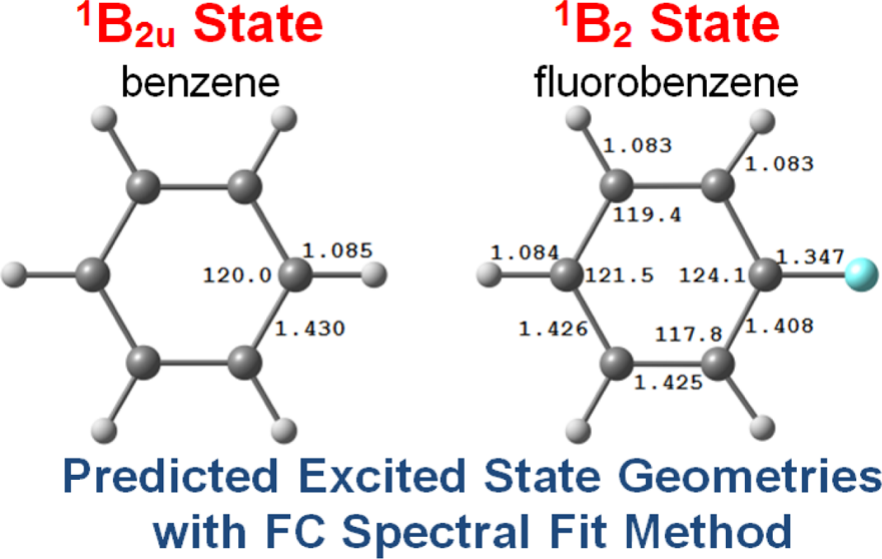

The parameters used in theoretical modeling of vibrational patterns within Franck–Condon (FC) approximation can be adjusted to match the vibrationally well-resolved experimental absorption spectrum of molecules. These simulation parameters can then be used to reveal the structural changes occurring between the initial and final states assuming the harmonic oscillator approximation holds for both states. Such a theoretical approach has been applied to benzene and fluorobenzene to disclose the first excited-state geometries of both compounds. The carbon–carbon bond length of benzene in the ^1^B_2u_ state has been calculated as 1.430 Å, which is in very good agreement with the experimental bond length of 1.432 Å. The FC spectral fit method has been exploited to reveal the ^1^B_2_ state of fluorobenzene as well. Commonly employed density functional theory (DFT) and time-dependent DFT methods have been used to calculate the ground- and excited-state geometries of both compounds, respectively. The comparison of geometrical parameters and vibrational frequencies at the relevant states shows that frequently used hybrid functionals perform quite well in the ground state, whereas their performances drop considerably while predicting the excited-state properties. Among the hybrid functionals studied, TD-B3LYP with 6-31+G(d) basis set can be chosen to calculate the excited-state properties of molecules, albeit with much less anticipation of accuracy from the performance that B3LYP usually shows at the ground state.

## 1 Introduction

The design of novel materials in optoelectronic applications requires good knowledge of excited-state properties.^1−3^ Theoretically speaking, it is a computationally demanding work since electron correlation plays an important role in such studies. From pioneering works of Hückel to semiempirical methods and lately to ab initio methods, a larger amount of electron correlation has been worked out to reveal the properties of the electronically excited states. Configuration interaction (CI) schemes,^4^ symmetry-adapted cluster CI,^5^ multireference approaches,^6,7^ various coupled-cluster (CC) techniques,^8−10^ and time-dependent Hartree–Fock approximation^11^ have been used in a broad range of problems in chemistry as well as in physics. In CC approximations (CCS, CC2, CCSD, CC3, and so on), the quality of excited-state potential surfaces of molecules has improved substantially toward the full CI limit.^12−14^ However, even with today’s computational resources, it is hard to apply such theories for medium- to large-sized molecules as the computational cost increases substantially with molecular size. For instance, the scaling of computational work in the CC series is *N*^4^ for CCS, *N*^5^ for CC2, *N*^6^ for CCSD, *N*^7^ for CC3, and so on (where *N* is the number of orbitals).

Density functional theory (DFT) methods have been vastly used in predicting the ground-state geometries of molecules and solids. Lately, time-dependent DFT (TD-DFT) methods have been exploited to reveal the excited-state properties of molecules at the computational price of Hartree–Fock (HF) or time-dependent HF calculations.^15,16^ Nonetheless, the accuracy of excited-state geometries predicted by TD-DFT methods is still an active area of research.^17−19^ There is a need for the experimental data set for excited-state geometries of molecules for benchmarking efforts. However, excited-state geometries of few molecules are measured experimentally, and such molecules are usually small in size.^20−23^ In general, the experimental investigation of structures and potential energy surfaces for excited states is often considerably more challenging than for ground states. Since there is a lack of experimental data on excited-state structures, several computational studies used the results obtained from computationally expensive methods on some molecules for evaluating the performance of various types of TD-DFT methods.^17,20,24^ This is, however, inherently problematic as there is no concrete evidence that such molecules possess the structures predicted by selected quantum mechanical methods. Besides, computationally demanding methods cannot be used on medium- to large-sized molecules due to the need for enormous computational power.

In our recent study, we have successfully shown that the excited-state geometries of relatively large molecules can be predicted by using Franck–Condon (FC) analysis of optical spectrum (FC spectral fit method) and then performing reverse engineering on the simulation parameters to extract information regarding the structural changes occurring upon photoexcitation.^25^ The only requirement for successful application of the FC spectral fit method is the need for availability of the vibrationally well-resolved optical spectrum of the molecule in question. In this regard, excited-state geometric parameters of naphthalene, perylene, and pyrene were successfully predicted due to vastly available experimental data reported on these polyaromatic hydrocarbons.^25^

**Figure 1:**
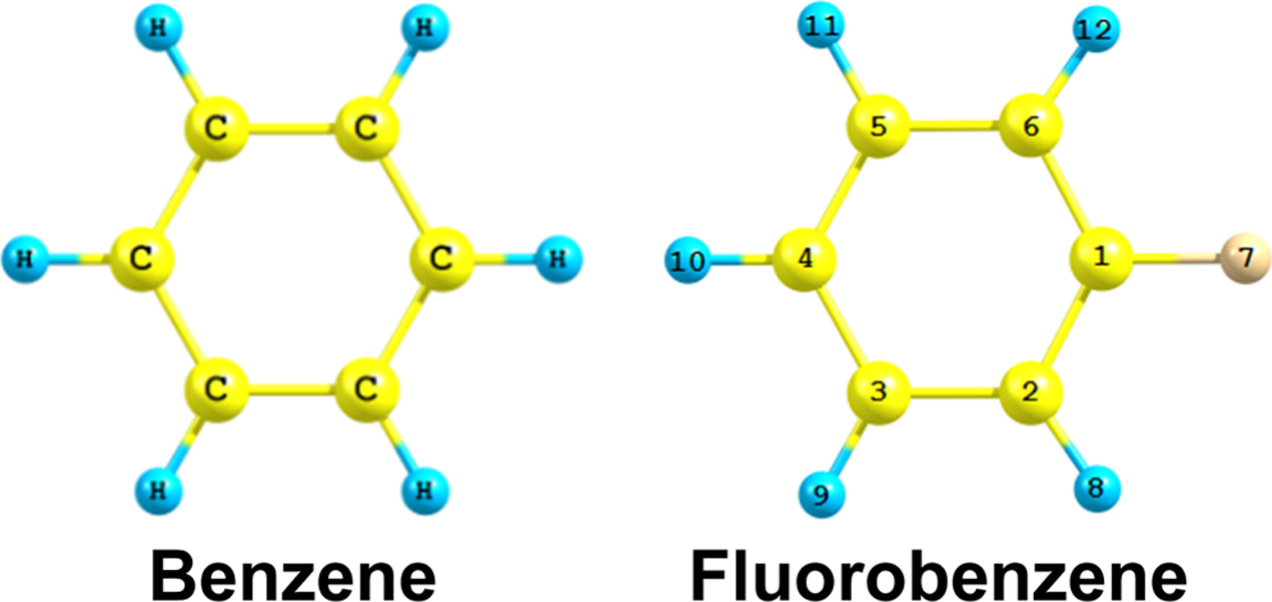
Molecular structure of studied molecules along with atom labeling/numbering of each atom.

In this work, we focused our attention on benzene and fluorobenzene (Figure 1), which are relatively smaller in size and showed that the FC spectral fit method works equally well for small molecules too. The predicted lowest excited-state geometries of both molecules were further used as reference in gauging the performance of commonly employed TD-DFT methods for excited-state geometries. There exist many experimental reports on vibrational frequencies as well as gas-phase UV/visible absorption spectra of these compounds, which make it possible to assess the quality of the calculated results with those of the experiment. All major vibrational FC progressions and their relative intensities have been identified for the normal modes that are involved in the spectra of both compounds. Hot bands are also identified and used to improve the spectral match between the simulated optical line shapes and the experimental ones.

## 2 Computational and Theoretical Methods

Optimized ground-state (excited-state) geometries of benzene and fluorobenzene were obtained by using B3LYP (TD-B3LYP), M06-2X (TD-M06-2X), PBEPBE (TD-PBEPBE), BMK (TD-BMK), wB97XD (TD-wB97XD), CAM-B3LYP (TD-CAM-B3LYP), and HF (CIS) methods, as implemented in the Gaussian 16 software package.^26^ All geometry and frequency calculations were performed with the 6-311+G(d,p) basis set unless otherwise noted. Frequency calculations were also performed to reveal the vibrational frequencies and normal modes for all structures with the selected theory and basis set. The absence of imaginary frequencies in the frequency calculations indicated that stable geometries were obtained for all the calculations presented in this work. Scaled vibrational frequencies (0.98 for the B3LYP functional) were used in the estimation of the excited-state geometries in relevant equations.^27^ TD-DFT calculations were performed on B3LYP-optimized geometries with the same basis set used in geometry optimizations.

The theoretical approach to predict the excited-state geometries of molecules has been given in detail in our previous publication.^25^ Therefore, only major steps are presented in this study. The following equation has been used to simulate the absorption line shapes within FC approximation
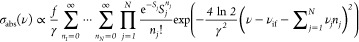
1In the above equation, *f* is the oscillator strength of relevant transition, γ is the linewidth of absorption lines which accounts for inhomogeneous broadening effects, *S*_*j*_ is the dimensionless Huang–Rhys factor for the mode *j* with frequency ν_j_, *N* is the number of vibrational modes with significant Huang–Rhys factors, *n*_*j*_ is the range of summation (0–5 for benzene, 0–3 for fluorobenzene), ν_if_ is the electronic origin (0–0 peak location), and ν is the wavenumber (cm^–1^). Huang–Rhys factor, *S*_*j*_, for mode *j* is evaluated as follows^28,29^
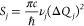
2where *ℏ* is the reduced Planck’s constant and *c* is the speed of light in the above equation. In order to calculate Huang–Rhys factor for each vibrational mode, Δ***Q*** must be calculated, which reflects the geometrical change between the ground-state and excited-state geometries.^28,30,31^
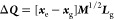
3Here, ***L***_g_ is the 3 N vector of the normal coordinates of the vibrational modes in the ground state, ***M*** is the 3 N × 3 N diagonal matrix of atomic masses, ***x***_e_ and ***x***_g_ are the 3 N dimensional vectors of Cartesian coordinates in the excited and ground states, respectively. After rearranging eq 3, ***x***_e_ can be expressed as^25^
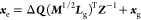
4where ***Z*** is
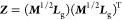
5

It is important to note that ***Z***^–1^ must be evaluated using the singular value decomposition technique as outlined in ref (25). The Duschinsky matrix (***J***) for fluorobenzene is obtained by multiplying matrices ***L***_g_^T^ and ***L***_e_ composed of a_1_ modes, which play major role in geometrical change in between the ground and the excited states.^30^

The major step in applying the FC spectral fit method is finding the Huang–Rhys factors and the associated frequencies that can best reproduce the experimental spectrum. In order to achieve this task, one needs to vary *S*_*j*_ of vibrational frequency ν_*j*_ in eq 1 and simulate the whole spectrum until a close match is obtained between the simulated line shapes and the experimental absorption intensities ( must be minimized). Once *S*_*j*_ values are empirically determined, the absolute values of Δ*Q*_*j*_ can be calculated with eq 2, and then, the Cartesian coordinates of the excited state, ***x***_e_, can be found by using eq 4. The normal modes calculated with quantum mechanical methods (in this case, the B3LYP/6-311+G(d,p) method) must be used in eq 3. During the survey of best spectral fit, it might be best if experimental ν_*j*_ is used for successful reproduction of the experimental spectrum. If experimental ν_*j*_ values are not known, then, the scaled frequencies obtained from the quantum mechanical method can be used. Within this context, the vibrational frequencies calculated with B3LYP or M06-2X^25^ should provide accurate enough frequency data that can be used in the simulation code. If the molecule is relatively large (that is, the vibrational frequencies do not change considerably between the ground-state and excited-state manifold), then the use of calculated ν_*j*_ for ground state should be sufficient for simulation efforts. On the other hand, for small molecules, the vibrational frequencies calculated at the excited state of interest should be utilized for simulation of the absorption spectrum. Both benzene and fluorobenzene have been widely studied in the literature;^32−39^ therefore, the reported experimental excited-state frequencies have been used in the simulations as detailed below.

## 3 Results and Discussion

### 3.1 Ground-State Geometric Properties

Ground-state geometries of benzene (^1^A_1g_) and fluorobenzene (^1^A_1_) were determined at various DFT levels as well as through the HF method. The reported literature studies including CAS (complete active space self-consistent field), CASPT2 (complete active space perturbation theory through second-order), and CCSD (coupled-clusters-singles-doubles) were also compared with the experimental bond lengths of benzene as given in Table 1. For benzene, the ground-state geometry has *D*_6h_ symmetry, whereas fluorobenzene adopts *C*_2V_ symmetry. Computationally demanding methods such as CAS, CASPT2, and CCSD yield very similar bond lengths to those observed in experimental studies for the ground state of benzene. CASPT2, however, stands out as the best performing method for structural parameters, displaying very good agreement with the experimental *R*_CC_ and *R*_CH_ bond lengths of benzene.

**Table 1:** Experimental and Calculated Bond Distances (Å) of Ground and Excited States (^1^B_2u_) of Benzene

		*R*_CC_	*R*_CH_
S_0_	Exp.^a^	1.397	1.082
	CAS(6,6)^b^	1.398	1.076
	CASPT2^c^	1.396	1.081
	CCSD^d^	1.392	1.079
	HF^e^	1.386	1.076
	B3LYP^e^	1.395	1.084
	M06-2X^e^	1.392	1.084
	PBEPBE^e^	1.401	1.093
	BMK^e^	1.397	1.086
	wB97XD^e^	1.392	1.084
	CAM-B3LYP^e^	1.389	1.084

		*R*_CC_	*R*_CH_
S_1_	Exp.^f^	1.432	1.084
	CAS(6,6)^b^	1.435	1.074
	CASPT2^c^	1.432	1.080
	CCSD^d^	1.425	1.078
	CIS^e^	1.413	1.074
	TD-B3LYP^e^	1.425	1.082
	TD-M06-2X^e^	1.422	1.082
	TD-PBEPBE^e^	1.433	1.091
	TD-BMK^e^	1.429	1.084
	TD-wB97XD^e^	1.420	1.082
	TD-CAM-B3LYP^e^	1.419	1.082
	**predicted from FC spectral fit**	**1.430**	**1.085**

a Reference (40).

b Reference (41) and the references cited therein.

c Reference (38) and the references cited therein.

d Reference (38) with the TZ2P basis set.

e This work with the 6-311+G(d,p) basis set.

f Reference (36).

Among DFT methods, B3LYP shows rather good performance for both benzene (Table 1) and fluorobenzene (Table 2), whereas the HF method underestimates C–C and C–H bond lengths considerably in both compounds. Range-corrected CAM-B3LYP slightly underestimates bond lengths for both molecules. The BMK optimized geometry of benzene has an *R*_CC_ of 1.397 Å, which is in perfect agreement with the experiment. However, the same functional shows rather poor performance in predicting the ground-state geometrical parameters of fluorobenzene. Apart from comparison of optimized geometric parameters, the close correlation of the calculated vibrational frequencies with those of the experiment suggests how well a particular method reflects the potential energy surface in the ground state. This is rather important because the normal modes of ground-state geometries will be used in prediction of excited-state geometries as a final state. Therefore, the correlation between the experimental and calculated frequencies has been used to judge the quality of a specific method in representing the ground-state manifold of studied molecules. For instance, the coefficient of determination (*R*^2^) between experimental ground-state frequencies and calculated frequencies with the B3LYP functional (Table 3) is 0.9999, suggesting a very good representation of ground-state properties in the ground state for benzene for the aforementioned hybrid functional. The R^2^ values for other methods are 0.9988 for HF, 0.9998 for M06-2X, 0.9997 for PBEPBE, 0.9996 for BMK, 0.9998 for wB97XD, and 0.9998 for CAM-B3LYP optimized geometries for benzene. The same *R*^2^ values from linear fit for fluorobenzene (Table 4) are 0.9998 for B3LYP, 0.9998 for M06-2X, 0.9995 for PBEPBE, 0.9998 for BMK, 0.9998 for wB97XD, 0.9998 for CAM-B3LYP, and 0.9995 for HF. Thus, the normal modes obtained through frequency calculation with the B3LYP functional have been chosen for subsequent use in vibronic spectrum simulation due to close estimation of structural parameters as well as good representation of the potential energy surface in the ground state of both molecules.

**Table 2:** Experimental and Calculated Bond Distances (Å) of Ground and Excited States (^1^B_2_) of Fluorobenzene

	Method	R (Å) C_1_F_7_	R (Å) C_1_C_2_	R (Å) C_2_C_3_	R (Å) C_3_C_4_	R (Å) C_2_H_8_	R (Å) C_3_H_9_	R (Å) C_4_H_10_	A (deg) F_7_C_1_C_2_	A (deg) C_6_C_1_C_2_	A (deg) C_1_C_2_C_3_	A (deg) C_2_C_3_C_4_	A (deg) C_3_C_4_C_5_	MAE (mÅ)
S_0_														
	**Exp.**^a^	**1.355**	**1.382**	**1.395**	**1.395**	**1.077**	**1.078**	**1.077**	**118.5**	**123.1**	**118.1**	**120.5**	**119.8**	
	B3LYP	1.357	1.386	1.394	1.395	1.083	1.084	1.083	118.7	122.6	118.3	120.5	119.8	3.6
	M06-2X	1.345	1.383	1.391	1.392	1.083	1.083	1.083	118.7	122.6	118.3	120.4	119.9	5.0
	PBEPBE	1.363	1.393	1.400	1.400	1.091	1.092	1.092	118.7	122.5	118.3	120.5	119.8	10.3
	BMK	1.341	1.389	1.397	1.397	1.084	1.085	1.085	118.7	122.6	118.3	120.5	119.8	7.1
	wB97XD	1.347	1.383	1.390	1.391	1.083	1.084	1.084	118.8	122.5	118.4	120.5	119.8	7.4
	CAM-B3LYP	1.351	1.380	1.389	1.389	1.082	1.083	1.083	118.7	122.7	118.3	120.4	119.6	4.9
	HF	1.328	1.377	1.386	1.386	1.074	1.075	1.074	118.8	122.4	118.4	120.5	119.7	8.4
**S**_**1**_														
	TD-B3LYP	1.342	1.416	1.424	1.424	1.081	1.081	1.083	117.6	124.8	117.2	119.4	122.0	3.0
	TD-M06-2X	1.330	1.413	1.419	1.422	1.080	1.081	1.083	117.5	125.0	117.1	119.3	122.1	5.4
	TD-PBEPBE	1.353	1.422	1.432	1.429	1.089	1.090	1.092	117.4	125.2	117.0	119.4	122.2	7.3
	TD-BMK	1.326	1.420	1.427	1.429	1.082	1.083	1.085	117.5	124.9	117.2	119.3	122.1	5.7
	TD-wB97XD	1.331	1.413	1.419	1.421	1.081	1.081	1.084	117.6	124.7	117.3	119.4	122.0	5.0
	TD-CAM-B3LYP	1.334	1.410	1.417	1.419	1.080	1.080	1.083	117.6	124.9	117.2	119.4	122.0	5.3
	CIS	1.313	1.406	1.410	1.416	1.072	1.072	1.074	117.9	124.2	117.6	119.5	121.5	13.3
	**predicted by FC spectral fit**	**1.347** (±0.002)	**1.408** (±0.002)	**1.425** (±0.001)	**1.426** (±0.002)	**1.083** (±0.001)	**1.083** (±0.001)	**1.084** (±0.001)	**117.9**	**124.1**	**117.8**	**119.4**	**121.5**	

a Reference (42).

**Table 3:** Vibrational Frequencies (cm^–1^) of the Ground and Excited States of Benzene

		S_0_			S_1_		
modes	Sym.	Exp.^a^	B3LYP^b^	BMK	Exp.^a^	TD-B3LYP	TD-BMK
1	a_1g_	993	1010 (990)	1008	923	954	955
2	a_1g_	3074	3191 (3127)	3208	3093	3216	3219
3	a_2g_	1350	1380 (1352)	1392	1327	1362	1364
4	b_2g_	707	719 (705)	726	365	329	304
5	b_2g_	990	1019 (999)	1048	745	792	811
6	e_2g_	608	622 (610)	624	521	530	525
7	e_2g_	3057	3165 (3102)	3183	3077	3189	3192
8	e_2g_	1600	1632 (1599)	1638	1516	1563	1574
9	e_2g_	1178	1197 (1173)	1208	1148	1177	1182
10	e_1g_	847	866 (849)	897	581	598	612
11	a_2u_	674	691 (677)	722	515	555	569
12	b_1u_	1010	1022 (1002)	1023		1001	1000
13	b_1u_	3057	3155 (3092)	3174		3185	3188
14	b_2u_	1310	1334 (1307)	1309	1570	1464	1518
15	b_2u_	1149	1174 (1151)	1176	1150	1174	1179
16	e_2u_	399	412 (404)	414	238	237	249
17	e_2u_	967	989 (969)	1019	717	754	780
18	e_1u_	1038	1058 (1037)	1060	920	966	975
19	e_1u_	1484	1509 (1479)	1518	1405	1443	1452
20	e_1u_	3065	3181 (3117)	3198	3084	3205	3208

a Reference (41) and the references cited therein.

b Values in parentheses are scaled by 0.98.

**Table 4:** Vibrational Frequencies (cm^–1^) of the Ground and Excited States of Fluorobenzene

		S_0_		S_1_	
modes	Sym.	Exp.^a^	B3LYP^b^	Exp.^c^	TD-B3LYP
1	a_1_	3094	3203 (3139)		3230
2	a_1_	3080	3191 (3127)		3213
3	a_1_	3061	3170 (3107)		3186
4	a_1_	1605	1633 (1600)		1559
5	a_1_	1500	1522 (1492)		1447
6	a_1_	1238	1234 (1209)	1230	1237
7	a_1_	1156	1174 (1151)		1149
8	a_1_	1023	1038 (1017)	917	987
9	a_1_	1009	1017 (997)	969	959
10	a_1_	809	818 (802)	765	793
11	a_1_	517	524 (514)	460	472
12	a_2_	957	975 (956)	643	635
13	a_2_	818	830 (813)	509	494
14	a_2_	414	422 (414)	206	168
15	b_1_	978	994 (974)	755	802
16	b_1_	895	909 (891)	661	679
17	b_1_	754	766 (751)	555	590
18	b_1_	687	688 (674)	451	475
19	b_1_	498	507 (497)	331	319
20	b_1_	233^c^	236 (231)	167	174
21	b_2_		3201 (3137)		3226
22	b_2_	3069	3179 (3115)		3206
23	b_2_	1605	1642 (1609)		1460
24	b_2_	1460	1485 (1455)		1420
25	b_2_	1301	1344 (1317)	1589	1523
26	b_2_		1324 (1298)		1300
27	b_2_	1128	1179 (1155)		1170
28	b_2_	1066	1089 (1067)	955	1011
29	b_2_	614	627 (614)	518	526
30	b_2_	400	404 (396)	388	392

a Reference (43).

b Values in parentheses are scaled by 0.98.

c Reference (33).

### 3.2 Vibronic Spectrum Simulation

The first excited state of benzene (^1^A_1g_ → ^1^B_2u_ transition) is symmetry-forbidden.^32,34^ However, such transition can still be observed due to Herzberg–Teller coupling, albeit with very low intensities.^41^ The intensity borrowing from the allowed transitions (^1^A_1g_ → ^1^E_1u_ and ^1^B_2u_ → ^1^E_2g_ transitions) has been well studied in the literature.^38,44^ Depending on the orientation of benzene relative to the electric polarization vector, the symmetry of the dipole moment matrix element can be either e_1u_ or a_2u_. The first term of the electronic transition is forbidden by symmetry; however, some higher-order terms can lead to allowed transitions when the symmetry of the modes combines as b_2u_⊗e_1u_⊗a_1g_ = e_2g_ or b_2u_⊗a_2u_⊗a_1g_ = b_1g_. Since there is no vibrational mode in benzene with b_1g_ symmetry, the in-plane e_2g_ modes will induce intensity in the absorption spectrum for the lowest excited state of benzene. The inducing modes for the allowed transition for the first excited state in the *D*_6h_ symmetry belong to doubly degenerate e_2g_ irreducible representation. These modes are ν_6_, ν_7_, ν_8_, and ν_9_, as listed in Table 3. The band origin (0–0 peak) cannot be observed due to one-photon forbidden symmetry of ^1^A_1g_ → ^1^B_2u_ transition. However, as mentioned above, the inducing modes should appear in the spectrum and can be seen as false origins since they do not originate from 0 to 0 transition. All of the peaks in the absorption spectrum of benzene should contain the vibrational modes of e_2g_ symmetry and their combinations with other modes.

**Figure 2:**
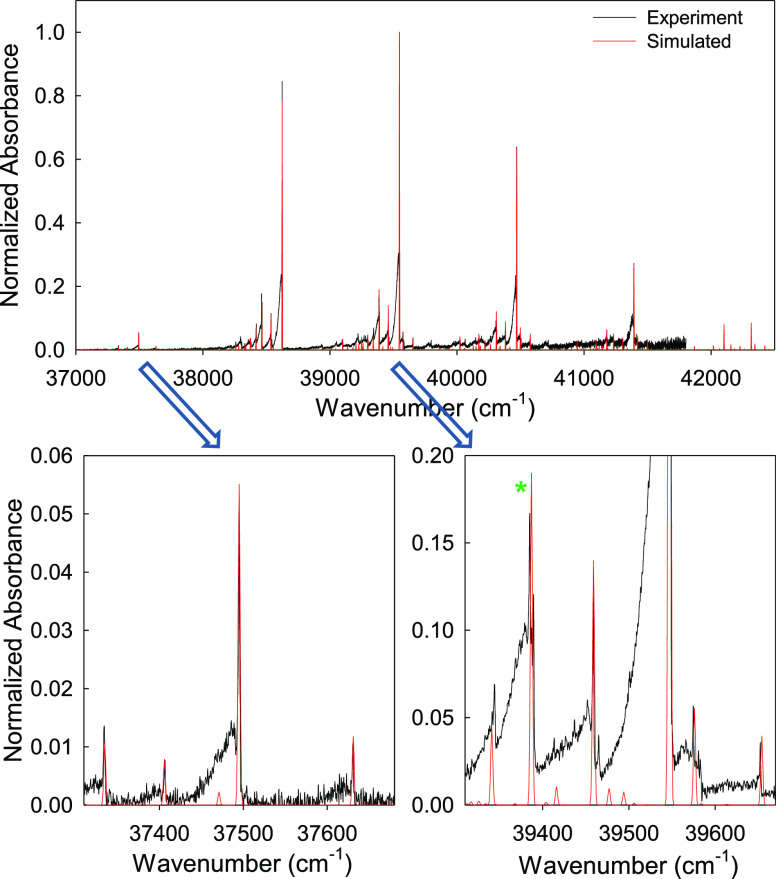
Simulated (red) and experimental (black, adapted with permission from ref (46), Copyright 2013 Creative Commons Attribution 4.0 License) absorption spectrum (^1^A_1g_ → ^1^B_2u_ transition) of benzene (top panel). Lower panels show the expanded areas of the simulated spectra with green asterisk for possible Fermi resonance splitting. The simulated spectrum has been obtained through the FC spectral fit method as detailed in the Computational and Theoretical Methods section.

After analyzing the experimental absorption spectrum^45^ of benzene for the ^1^B_2u_ state (Figure 2, black line), it is clear that the major progression in the spectrum has originated from ν_6_ (squashing mode). There are selection rules that need to be followed when building up the vibronic progressions of ^1^A_1g_ → ^1^B_2u_ transition.^41^ For the e_2g_ inducing modes, Δν should be equal to ±1. For totally symmetric a_1g_ and a_2g_ modes, Δν should be equal to 0, ±1, ±2, ±3,..., whereas all the other modes have a selection rule that satisfies the condition Δν = 0, ±2, ±4, ±6,.... When *T* > 0, some low frequency modes can be populated and therefore hot bands will be observed in the experimental spectrum. Primary modes with appreciable intensities that would lead to hot bands are e_2u_ (399 cm^–1^ in the ground state and 238 cm^–1^ in the excited state) and a_2u_ (674 cm^–1^ in the ground state and 515 cm^–1^ in the excited state) modes. Other modes and transitions are excluded from the simulation to save computational time. Besides, the purpose of this study is to reveal the geometry change between the ground state and the excited state for which only totally symmetric modes matter at this stage.

The convention for showing the vibrational progressions is as follows: a mode is represented by its number and the number of vibrational excitation in the lower or upper state as a subscript or a superscript, respectively. All the major identified modes are listed in Table 5, and the computed spectrum is given in Figure 2 (red line). The simulated spectrum consists of major peaks and can be seen as combination of fundamentals, overtones, combinations, and hot bands of major peaks. The formula given in eq 1 can be used for the allowed one-photon absorption spectrum. Since the first excited state of benzene is formally forbidden and is observed only through inducing modes, each vibrational progression is simulated separately and then summed up to obtain the total simulated spectrum. 6_0_^1^ progression has the highest contribution to the overall spectrum; therefore, the highest peak in that progression is normalized to 1. The other progressions were scaled (6_1_^2^ with 0.14, 6_1_^0^ with 0.07, 6_2_^1^ with 0.01, 9_0_^1^ with 0.05, and 7_0_^1^ with 0.08) to match the experimental intensities after the simulation of each progression separately. As emphasized above, the progression 6_0_^1^ has been found to carry most of the vibrational progression between the ground state and the excited state, as also reported by other researchers.^38,41^Table 5 compiles the experimental relative intensities of vibrational modes and their corresponding assignments based on the simulated spectrum. As discussed in the Computational and Theoretical Methods section, the experimental frequencies have been used in the simulation of the spectrum in order to precisely unveil the major modes and their relative intensities.

**Table 5:** Assigned Experimental Frequencies (cm^–1^) and Relative Intensities of the Main Vibronic Bands of the ^1^A_1g_ → ^1^B_2u_ Transition in Benzene

frequency (cm^–1^)	relative intensity	assignment	frequency (cm^–1^)	relative intensity	assignment
37,334	0.014	6_1_^0^16_1_^1^	39,228	0.031	6_0_^1^1_0_^1^16_2_^2^
37,406	0.008	6_2_^1^	39,249	0.031	9_0_^1^
37,495	0.051	6_1_^0^	39,253	0.036	6_2_^1^1_0_^2^
37,630	0.011	6_0_^1^1_1_^0^	39,343	0.069	6_1_^0^1_0_^2^
38,103	0.000	0–0	39,388^a^	0.167	6_0_^1^1_0_^1^16_1_^1^
38,217	0.015	6_1_^2^16_2_^2^	39,458	0.119	6_1_^2^1_0_^1^
38,259	0.025	6_1_^0^1_0_^1^16_1_^1^	39,548	1.000	6_0_^1^1_0_^1^
38,282	0.042	6_0_^1^4_1_^1^	39,574	0.057	6_0_^1^16_0_^4^
38,303	0.020	6_0_^1^16_2_^2^	39,652	0.036	6_0_^1^11_0_^2^
38,305	0.025	6_0_^1^11_2_^2^	40,024	0.032	6_0_^1^1_0_^1^16_0_^2^
38,330	0.020	6_2_^1^1_0_^1^	40,147	0.039	6_0_^1^1_0_^2^16_2_^2^
38,378	0.024	6_1_^2^16_1_^1^	40,173	0.045	9_0_^1^1_0_^1^
38,420	0.082	6_1_^0^1_0_^1^	40,263	0.043	6_1_^0^1_0_^3^
38,460	0.178	6_0_^1^16_1_^1^	40,312	0.093	6_0_^1^1_0_^2^16_1_^1^
38,465	0.150	6_0_^1^11_1_^1^	40,379	0.060	6_1_^2^1_0_^2^
38,536	0.114	6_1_^2^	40,470	0.639	6_0_^1^1_0_^2^
38,624	0.845	6_0_^1^	40,504	0.051	6_0_^1^1_0_^1^16_0_^4^
39,051	0.027	6_1_^2^16_0_^2^	40,573	0.034	6_0_^1^1_0_^1^11_0_^2^
39,101	0.032	6_0_^1^16_0_^2^	41,179	0.062	7_0_^1^
39,183	0.021	6_1_^0^1_0_^2^16_1_^1^	41,391	0.272	6_0_^1^1_0_^3^

a Possible Fermi resonance splitting due to combination bands involving 16_1_^1^ and 11_1_^1^.

In the simulated spectrum, 6_2_^1^ progression has the 1_0_^*n*^ (*n* = 0–2) sequence; 6_1_^0^ progression has 1_0_^*n*^ (*n* = 0–3) and 16_*n*_^*n*^ (*n* = 0–1) sequences and their combinations; 6_1_^2^ progression has 1_0_^*n*^ (*n* = 0–3), 16_*n*_^*n*^ (*n* = 0–1), and 16_0_^2*n*^ (*n* = 0–1) sequences and their combinations; 6_0_^1^ progression has 1_0_^*n*^ (*n* = 0–4), 16_*n*_^*n*^ (*n* = 0–2), 16_0_^2*n*^ (*n* = 0–2), 1_1_^*n*^ (*n* = 0–1), 11_0_^2*n*^ (*n* = 0–1) sequences and their combinations; 9_0_^1^ progression has the 1_0_^*n*^ (*n* = 0–1) sequence; and 7_0_^1^ progression has the 1_0_^*n*^ (*n* = 0–1) sequence. There are very low intensity sequences that could be used to improve the results; however, they are neglected and are not of interest for the present purposes of this work. The lower panels in Figure 2 nicely illustrate that all major peaks have been reproduced and identified in the gas phase experimental absorption spectrum of benzene. However, possible Fermi resonance splitting has been observed at ∼39,385 cm^–1^, most probably due to mixing of combination bands involving 16_1_^1^ and 11_1_^1^ and hence causing some mismatches at relative positions of some low intensity peaks between the simulated and the experimental spectrum.

The experimental spectrum in Figure 2 is adopted from ref (46). The γ value for line broadening was selected as 2 cm^–1^ for simulated absorption line shapes. Due to the symmetry of the benzene molecule, the transition dipole moment vanishes as expected, and the 0–0 peak is not observed. The main peaks in the spectrum are from the progression involving the totally symmetric mode ν_1_, whose Huang–Rhys factor is 1.27 and induced by the promoting mode ν_6_. We have not found any peak that would fit to totally symmetric mode ν_2_ (C–H stretching modes), induced by promoting mode ν_6_. The experimental data is not available beyond ∼41,800 cm^–1^. Therefore, such a search for other inducing modes (ν_7_, ν_8_, and ν_9_) was not possible for totally symmetric mode ν_2_. However, it is clear that other inducing modes contribute quite less to the overall intensity of absorption line shapes for the totally symmetric mode ν_1_ (see relative intensities listed in Table 5). Thus, Huang–Rhys factor for mode ν_2_ is assumed to be zero, in accordance with a very small Huang–Rhys factor of 0.0090 obtained with the use of a quantum mechanically optimized ground and excited-state geometries of benzene.^41^ As a result, Huang–Rhys factor with a value of 1.27 (for totally symmetric mode ν_1_) predicted from FC spectral fit is used only in eq 4 to reveal the ^1^B_2u_ excited-state geometry of benzene. The differences in between the bond lengths upon going from the ground state to the excited state are calculated as 0.0333 Å for *R*_CC_ and 0.0028 Å for *R*_CH_. Callomon et al. estimated 0.038 Å increase for *R*_CC_ upon photoexcitation based on analysis of rotational constants,^44^ in close alignment with the prediction presented in this study. If the experimental ground-state equilibrium geometry (Table 1) of benzene is used as the reference structure (***x***_g_),^40^ then the predicted ^1^B_2u_ state *R*_CC_ and *R*_CH_ bond lengths from the FC spectral fit method are found to be 1.430 and 1.085 Å, respectively. These numbers are very close to the experimental bond lengths of 1.432 Å (±1 mÅ) and 1.084 Å (±1 mÅ) in the excited state.^36^ Therefore, one can argue that the excited-state geometric parameters obtained through the FC spectral fit method are quite reliable for benzene, as also verified in a previous study for prediction of excited-state geometries of larger polyaromatic hydrocarbons.^25^

Fluorobenzene is the prototypical benzene derivative. Fluorobenzene is arguably least likely to deviate substantially from benzene in its vibrational behavior and its response to photoexcitation. However, the presence of fluorine has profound consequences for the spectroscopic properties of fluorobenzene through the resultant lowering of symmetry and the effect of lowering symmetry on the optical selection rules. Fluorobenzene belongs to the *C*_2V_ point group, and the lowest excited state (^1^A_1_ → ^1^B_2_ transition) is not symmetry forbidden as opposed to benzene (the corresponding transition is ^1^A_1g_ → ^1^B_2u_).^47^ Nonetheless, the calculated oscillator strength of ^1^A_1_ → ^1^B_2_ transition is rather small (*f* = 0.0132), in accordance with the experimental observations.^48^ However, eq 1 can still be used in optical spectrum simulation due to one-photon allowed nature of photoexcitation for the relevant state. The normal modes of fluorobenzene are divided into four irreducible representations, comprising 11 a_1_ modes, 10 b_2_ modes, 6 b_1_ modes, and 3 a_2_ modes. The a_1_ and b_2_ modes are in-plane vibrations, and only a_1_ modes take part in ^1^A_1_ → ^1^B_2_ electronic transition, which is under investigation here. A majority of the 30 fundamental frequencies of fluorobenzene both in the ground and lowest excited state (^1^B_2_ state) have been successfully assigned in several literature studies.^33,39,43,48^

The experimental absorption spectrum of fluorobenzene has been taken from elsewhere.^48^ In order to match the experimental spectrum, ν_*if*_ is chosen as 37,820 cm^–1^ in the simulated spectrum. At the outset, the simulation was run for the involved a_1_ modes without inclusion of hot bands. However, the mismatch between the simulated line shapes and experimental ones required the usage of hot bands that are clearly present in the experimental spectrum. The vibrational frequencies and the empirically determined Huang–Rhys factors used in the simulated spectrum are ν_11_ (460 cm^–1^, *S*_11_ = 0.02), ν_10_ (765 cm^–1^, *S*_10_ = 0.34), ν_8_ (917 cm^–1^, *S*_8_ = 0.31), ν_9_ (969 cm^–1^, *S*_9_ = 0.38), ν_6_ (1230 cm^–1^, *S*_6_ = 0.11), and ν_5_ (1430 cm^–1^, *S*_5_ = 0.03). Note that the experimental S_1_ frequency of mode ν_5_ has not been reported before but found to be around 1430 cm^–1^ after varying the simulation parameters that could give the best match to the experimental spectrum. The hot bands used in the simulation code had frequencies entered as −66 cm^–1^ (ν_20_: from 233 cm^–1^ in S_0_ to 167 cm^–1^ in S_1_) with an *S* = 0.41 and −208 cm^–1^ (ν_14_: from 414 cm^–1^ in S_0_ to 206 cm^–1^ in S_1_), with an *S* = 0.17 and −309 cm^–1^ (ν_13_: from 818 cm^–1^ in S_0_ to 509 cm^–1^ in S_1_), and with an *S* = 0.05. It is important to mention that while Huang–Rhys factors for a_1_ modes can be considered to be a reflection of the magnitudes of vibronic coupling integrals for the associated modes between the initial and the final states, the same cannot be said for *S* values used for hot bands. This is because empirically determined Huang–Rhys factors for hot bands also incorporate the effects of thermal population of the relevant vibrational modes at *T* > 0. Therefore, the relative magnitudes of Huang–Rhys factors used in the simulation of hot bands should be interpreted with caution. It is also possible that there are more hot bands that have not been accounted for within the simulation parameters. Since the involved a_1_ modes are later on utilized to extract the excited-state geometry parameters, the majority of focus is therefore conveyed to the proper determination of Huang–Rhys factors of those modes in the vibronically resolved simulation of the fluorobenzene absorption spectrum.

**Figure 3:**
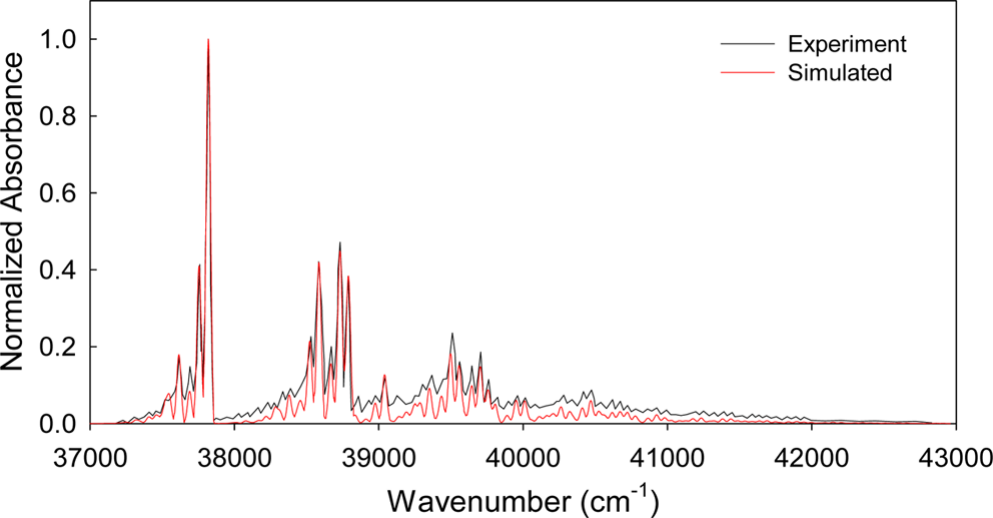
Simulated (red) and experimental^48^ (black, adapted with permission from ref (48), Copyright 2016 AIP Publishing License) absorption spectrum (^1^A_1_ → ^1^B_2_ transition) of fluorobenzene. The simulated peaks are broadened with γ = 35 cm^–1^ to match the experimental line shapes. The simulated spectrum has been obtained through the FC spectral fit method as detailed in the Computational and Theoretical Methods section.

The simulated spectrum of fluorobenzene is strikingly similar to the experimental one (Figure 3). Indeed, all major vibronic line shapes have been successfully reproduced with the chosen Huang–Rhys factors for the relevant modes. The slight intensity mismatch at higher wavenumbers can be associated to the relative crudeness of the employed method, as also has been observed in the simulated spectra of larger polyaromatic hydrocarbons.^25^

The empirically determined Huang–Rhys factors have been used in eq 2 to estimate |Δ*Q*| for the associated modes calculated with the B3LYP/6-311+G(d,p) method. The sign of Δ*Q*_*j*_ is a major concern since knowing *S*_*j*_ beforehand only helps in the determination of the absolute value of Δ*Q*_*j*_.^25^ Therefore, a survey of possible geometries, which resemble most the quantum mechanically calculated geometries, has been conducted and the signs of Δ*Q*_11_, Δ*Q*_10_, Δ*Q*_8_, Δ*Q*_9_, Δ*Q*_6_, and Δ*Q*_5_ are determined as +, +, +, – , +, and −, respectively. By inserting the estimated Δ*Q*_*j*_ in matrix form into eq 4, the excited-state geometry of fluorobenzene at the ^1^B_2_ state has been successfully predicted, and the relevant geometrical parameters are listed in Table 2. The maximum error for bond lengths is found to be at most 2 mÅ assuming that there exists an error range of ±0.02 for empirically determined *S*_*j*_ values.

**Figure 4:**
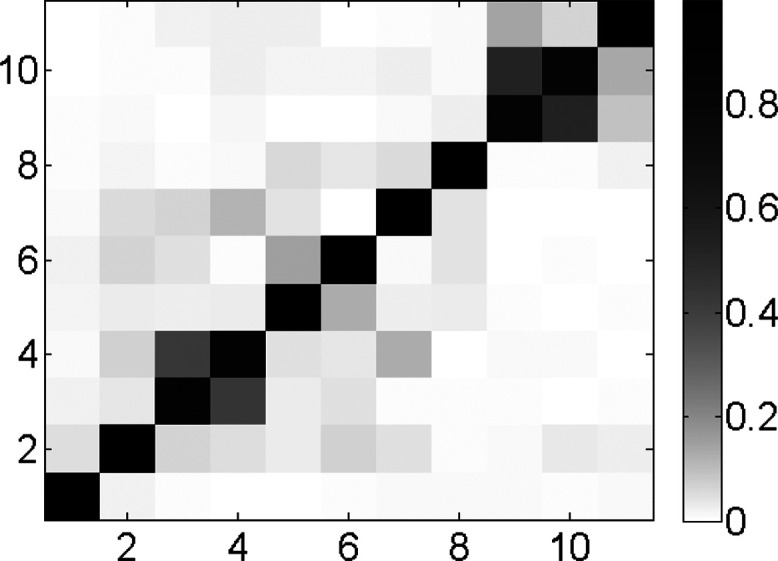
Graphical representation of the Duschinsky matrix ***J*** for the ^1^A_1_ → ^1^B_2_ transition of fluorobenzene for a_1_ modes with increasing frequencies (i.e., mode number 1 in the graph has the lowest vibrational frequency and mode number 11 has the highest vibrational frequency (see Table 5)). A shade of gray is associated with each element (i,j) in the figure based on the equivalence (0, white; 1, black).

The use of eq 1 requires that Duschinsky mixing is negligible between the ground-state and the excited-state normal modes.^30^ The ***L***_g_^T^ matrix, composed of a_1_ modes, was acquired from the frequency calculations performed on B3LYP optimized ground-state geometry. Similarly, the ***L***_e_ matrix, composed of a_1_ modes for excited-state geometry predicted through the FC spectral fit method, was generated from the frequency calculations with the TD-B3LYP method. It is clear that Duschinsky mixing (Figure 4) can be neglected for fluorobenzene due to insignificant mixing between the normal modes, and thus, the use of eq 1 is further confirmed with this result.

### 3.3 Performance of DFT Methods for Excited-State Geometries

The experimental excited-state (^1^B_2u_) geometry of benzene has been reported before^36^ and in very good agreement with the geometry predicted by using the FC spectral fit method. As far as we know, the experimental ^1^B_2_ state excited-state geometry of fluorobenzene has not been reported in the literature; therefore, the excited-state geometry predicted by the FC spectral fit method has been used as a reference for evaluation of performance of computational methods for excited states. Cvitaš et al. predicted that C–F bond decreases by either 0.009 Å or 0.028 Å, upon fitting rotational constant data with different methods.^35^ The geometry predicted by the FC spectral fit method shows that the C–F bond length decreases by 0.008 Å (see Table 2), in very good agreement with the change for the experimentally reported C–F bond length.

It is clear that the bond lengths calculated with the CIS method are too short for benzene, whereas CASPT2 again provides accurate excited-state geometrical parameters among the ab initio methods (Table 1). TD-BMK also performs well in predicting the *R*_CC_ bond length of benzene in the excited state. TD-B3LYP is the second best performing method in predicting the structural parameters of benzene at the ^1^B_2u_ state. Nonetheless, the *R*^2^ value for correlation of TD-BMK frequencies with the experimental frequencies is 0.9985, while the same number is 0.9981 for frequencies calculated at the TD-B3LYP level. Other functionals also possess similar or worse *R*^2^ values, suggesting that the excited-state potential energy surface predicted by TD-DFT methods are not as good as those calculated in the ground state. Specifically speaking, the mode ν_14_ (1570 cm^–1^) with b_2u_ symmetry deviates considerably from the calculated frequencies with TD-DFT methods. The origin of this deviation is not clear but the vibrational frequencies calculated with CC2 and CCSD methods appear to be more accurate (after scaling), as shown in the literature.^32^

The mean absolute errors (MAEs) for bond length differences in fluorobenzene are given in the last column of Table 2 for all functionals. The MAE for TD-BMK for excited-state geometry of fluorobenzene is 5.7 mÅ, and its performance exceeded by TD-B3LYP with an MAE of just 3.0 mÅ. CIS gives the worst performance and should be avoided in the calculation of excited-state geometries. On the other hand, for excited-state geometry of fluorobenzene almost all methods provides comparatively less reliable C–C and C–F bond lengths. That is, especially the accuracies of DFT methods for excited-state geometries are not very good, considering the wide variation and deviation of bond length data with the reference values. This observation is also validated by examining *R*^2^ values (0.9931 for TD-B3LYP, 0.9906 for TD-M06-2X, 0.9909 for PBEPBE, 0.9915 for BMK, 0.9936 for wB97XD, 0.9935 for CAM-B3LYP, and 0.9928 for CIS) calculated for correlation of the calculated excited-state frequencies with those of the experiment. As a result, one can conclude that the performances of TD-DFT methods for excited states are not particularly impressive, and yet, TD-B3LYP could serve better than other methods for such calculations.

One might argue that the effect of basis set sizes should be analyzed before reaching a definite conclusion. Tables 6 and 7 show the influence of various basis sets on structural parameters calculated with TD-B3LYP for benzene and fluorobenzene, respectively. The effect of basis set sizes on the calculated results is relatively small with decent size basis sets for both ground and excited states. Nevertheless, the TD-B3LYP method with 6-31+G(d,p) method appears to yield the lowest MAE values for both compounds in the excited state and can be promoted to be used in the calculation of excited-state geometries molecules or at least structurally similar polyaromatic hydrocarbons.

**Table 6:** Calculated Bond Distances of Ground and Excited State (^1^B_2u_) Structures of Benzene with the B3LYP Hybrid Functional along with Various Basis Sets

		*R*_CC_	*R*_CH_
S_0_	Exp.^a^	1.397	1.082
	6-31G(d)	1.397	1.087
	6-31+G(d)	1.399	1.087
	6-31G(d,p)	1.396	1.086
	6-31+G(d,p)	1.398	1.086
	6-311G	1.398	1.082
	6-311G(d,p)	1.394	1.084
	6-311+G(d,p)	1.395	1.085
	6-311+G(3df,2p)	1.391	1.082
	TZVP	1.392	1.084
	cc-PVTZ	1.391	1.082
	aug-cc-PVTZ	1.391	1.082

		*R*_CC_	*R*_CH_
S_1_	Exp.^b^	1.432	1.084
	6-31G(d)	1.428	1.085
	6-31+G(d)	1.429	1.085
	6-31G(d,p)	1.428	1.084
	6-31+G(d,p)	1.429	1.084
	6-311G	1.431	1.081
	6-311G(d,p)	1.425	1.082
	6-311+G(d,p)	1.425	1.082
	6-311+G(3df,2p)	1.421	1.080
	TZVP	1.423	1.081
	cc-PVTZ	1.421	1.080
	aug-cc-PVTZ	1.421	1.080
	**predicted by FC spectral fit**	**1.430**	**1.085**

a Reference (40).

b Reference (36).

**Table 7:** Calculated Bond Distances and Angles of Ground- and Excited-State (^1^B_2_) Structures of Fluorobenzene with the B3LYP Hybrid Functional along with Various Basis Sets

	method	R (Å) C_1_F_7_	R (Å) C_1_C_2_	R (Å) C_2_C_3_	R (Å) C_3_C_4_	R (Å) C_2_H_8_	R (Å) C_3_H_9_	R (Å) C_4_H_10_	A (deg) F_7_C_1_C_2_	A (deg) C_6_C_1_C_2_	A (deg) C_1_C_2_C_3_	A (deg) C_2_C_3_C_4_	A (deg) C_3_C_4_C_5_	MAE (mÅ)
S_0_														
	**Exp.**^a^	**1.355**	**1.382**	**1.395**	**1.395**	**1.077**	**1.078**	**1.077**	**118.5**	**123.1**	**118.1**	**120.5**	**119.8**	
	6-31G(d)	1.352	1.390	1.396	1.397	1.085	1.087	1.086	118.8	122.3	118.5	120.4	119.8	5.7
	6-31+G(d)	1.361	1.390	1.398	1.399	1.086	1.087	1.086	118.6	122.7	118.2	120.5	119.9	6.9
	6-31G(d,p)	1.351	1.390	1.396	1.397	1.085	1.086	1.086	118.8	122.3	118.5	120.5	119.8	5.7
	6-31+G(d,p)	1.361	1.390	1.398	1.398	1.085	1.086	1.086	118.6	122.7	118.2	120.5	119.9	6.4
	6-311G	1.403	1.387	1.398	1.399	1.080	1.081	1.081	118.5	123.1	118.1	120.3	120.0	10.0
	6-311G(d,p)	1.353	1.387	1.393	1.394	1.083	1.084	1.083	118.9	122.3	118.5	120.4	119.8	4.0
	6-311+G(d,p)	1.357	1.386	1.394	1.395	1.083	1.084	1.083	118.7	122.6	118.3	120.5	119.8	3.6
	6-311+G(3df,2p)	1.351	1.383	1.391	1.391	1.081	1.082	1.081	118.7	122.6	118.3	120.6	119.7	3.6
	TZVP	1.355	1.384	1.392	1.392	1.082	1.083	1.082	118.7	122.5	118.4	120.4	119.9	3.3
	cc-PVTZ	1.351	1.384	1.390	1.391	1.081	1.082	1.081	118.8	122.4	118.4	120.4	119.8	3.9
	aug-cc-PVTZ	1.353	1.383	1.391	1.391	1.080	1.081	1.081	118.7	122.5	118.4	120.4	119.9	3.0
S_1_														
	6-31G(d)	1.342	1.419	1.428	1.426	1.083	1.084	1.086	117.7	124.7	117.3	119.4	122.0	3.1
	6-31+G(d)	1.347	1.419	1.427	1.428	1.083	1.084	1.086	117.6	124.9	117.2	119.4	122.0	2.6
	6-31G(d,p)	1.342	1.419	1.427	1.426	1.082	1.083	1.085	117.7	124.7	117.3	119.4	122.0	2.9
	6-31+G(d,p)	1.347	1.419	1.427	1.428	1.083	1.083	1.085	117.5	124.9	117.1	119.4	122.0	2.3
	6-311G	1.393	1.414	1.431	1.430	1.078	1.079	1.081	117.5	125.0	117.1	119.5	121.7	10.6
	6-311G(d,p)	1.342	1.416	1.425	1.424	1.080	1.081	1.083	117.7	124.7	117.3	119.4	122.0	3.0
	6-311+G(d,p)	1.342	1.416	1.424	1.424	1.081	1.081	1.083	117.6	124.8	117.2	119.4	122.0	3.0
	6-311+G(3df,2p)	1.335	1.413	1.420	1.420	1.078	1.079	1.081	117.6	124.9	117.1	119.4	122.0	5.7
	TZVP	1.341	1.414	1.422	1.422	1.079	1.080	1.082	117.6	124.8	117.2	119.4	122.0	4.0
	cc-PVTZ	1.338	1.413	1.421	1.420	1.078	1.079	1.081	117.6	124.8	117.2	119.3	122.1	5.1
	aug-cc-PVTZ	1.338	1.413	1.420	1.420	1.078	1.079	1.081	117.6	124.8	117.2	119.3	122.1	5.3
	**predicted by FC spectral fit**	**1.347** (±0.002)	**1.408** (±0.002)	**1.425** (±0.001)	**1.426** (±0.002)	**1.083** (±0.001)	**1.083** (±0.001)	**1.084** (±0.001)	**117.9**	**124.1**	**117.8**	**119.4**	**121.5**	

a Reference (42).

## 4 Conclusions

In summary, the spectral empirical fitting approach with the use of FC approximation is an effective method to extract information regarding the excited-state geometries of molecules as long as well-resolved optical spectra are available to use for the relevant state. For both benzene and fluorobenzene, the optical spectra for the lowest excited states were simulated, and all major vibronic lines and hot bands were successfully reproduced. The Huang–Rhys factors used in the spectral simulation were exploited to predict the excited-state geometries, and an almost perfect agreement was achieved with the results obtained with the FC spectral fit method and the reported experimental results. The comparison of excited-state geometries calculated with commonly used DFT functionals with those predicted with the FC spectral fit method showed that TD-B3LYP performs better than other hybrid functionals. Nonetheless, the accuracies of excited-state geometries predicted by TD-DFT are not superior to their performances in the ground state. This has been shown by not only the assessment of geometrical parameters with experiment but also by comparison of vibrational frequencies for the relevant excited states with the experimental findings. It is clear that potential energy surfaces predicted by DFT methods at the excited state are not as good as their ground-state counterparts, with B3LYP being no exception to this observation.

## References

[ref1] Laquai F.; Park Y. S.; Kim J. J.; Basché T. Excitation Energy Transfer in Organic Materials: From Fundamentals to Optoelectronic Devices. Macromol. Rapid Commun. 2009, 30, 1203–1231. 10.1002/marc.200900309. 21638374

[ref2] Scheblykin I. G.; Yartsev A.; Pullerits T.; Gulbinas V.; Sundström V. Excited state and charge photogeneration dynamics in conjugated polymers. J. Phys. Chem. B 2007, 111, 6303–6321. 10.1021/jp068864f. 17521181

[ref3] Zhou P. W.; Han K. Unraveling the Detailed Mechanism of Excited-State Proton Transfer. Acc. Chem. Res. 2018, 51, 1681–1690. 10.1021/acs.accounts.8b00172. 29906102

[ref4] Head-Gordon M.; Rico R. J.; Oumi M.; Lee T. J. A Doubles Correction to Electronic Excited-States from Configuration-Interaction in the Space of Single Substitutions. Chem. Phys. Lett. 1994, 219, 21–29. 10.1016/0009-2614(94)00070-0.

[ref5] Nakatsuji H.; Hirao K. Cluster Expansion of Wavefunction - Pseudo-Orbital Theory Applied to Spin Correlation. Chem. Phys. Lett. 1977, 47, 569–571. 10.1016/0009-2614(77)85042-2.

[ref6] Grimme S.; Waletzke M. Multi-reference Moller-Plesset theory: computational strategies for large molecules. Phys. Chem. Chem. Phys. 2000, 2, 2075–2081. 10.1039/b000177p.

[ref7] Szalay P. G.; Müller T.; Lischka H. Excitation energies and transition moments by the multireference averaged quadratic coupled cluster (MR-AQCC) method. Phys. Chem. Chem. Phys. 2000, 2, 2067–2073. 10.1039/b000224k.

[ref8] Dalgaard E.; Monkhorst H. J. Some Aspects of the Time-Dependent Coupled-Cluster Approach to Dynamic-Response Functions. Phys. Rev. A 1983, 28, 1217–1222. 10.1103/physreva.28.1217.

[ref9] Koch H.; Jo/rgensen P. Coupled Cluster Response Functions. J. Chem. Phys. 1990, 93, 3333–3344. 10.1063/1.458814.

[ref10] Kowalski K.; Piecuch P. New coupled-cluster methods with singles, doubles, and noniterative triples for high accuracy calculations of excited electronic states. J. Chem. Phys. 2004, 120, 1715–1738. 10.1063/1.1632474. 15268302

[ref11] Bonche P.; Koonin S.; Negele J. W. One-Dimensional Nuclear Dynamics in Time-Dependent Hartree-Fock Approximation. Phys. Rev. C: Nucl. Phys. 1976, 13, 1226–1258. 10.1103/physrevc.13.1226.

[ref12] Császár A. G.; Allen W. D.; Schaefer H. F. In pursuit of the ab initio limit for conformational energy prototypes. J. Chem. Phys. 1998, 108, 9751–9764. 10.1063/1.476449.

[ref13] Evangelista F. A.; Allen W. D.; Schaefer H. F. High-order excitations in state-universal and state-specific multireference coupled cluster theories: Model systems. J. Chem. Phys. 2006, 125, 154113 10.1063/1.2357923. 17059245

[ref14] Feller D.; Peterson K. A.; Crawford T. D. Sources of error in electronic structure calculations on small chemical systems. J. Chem. Phys. 2006, 124, 054107 10.1063/1.2137323. 16468851

[ref15] Muniz-Miranda F.; Pedone A.; Battistelli G.; Montalti M.; Bloino J.; Barone V. Benchmarking TD-DFT against Vibrationally Resolved Absorption Spectra at Room Temperature: 7-Aminocoumarins as Test Cases. J. Chem. Theory Comput. 2015, 11, 5371–5384. 10.1021/acs.jctc.5b00750. 26574327

[ref16] Laurent A. D.; Jacquemin D. TD-DFT benchmarks: A review. Int. J. Quant. Chem. 2013, 113, 2019–2039. 10.1002/qua.24438.

[ref17] Bousquet D.; Fukuda R.; Maitarad P.; Jacquemin D.; Ciofini I.; Adamo C.; Ehara M. Excited-State Geometries of Heteroaromatic Compounds: A Comparative TD-DFT and SAC-CI Study. J. Chem. Theory Comput. 2013, 9, 2368–2379. 10.1021/ct400097b. 26583727

[ref18] Guido C. A.; Knecht S.; Kongsted J.; Mennucci B. Benchmarking Time-Dependent Density Functional Theory for Excited State Geometries of Organic Molecules in Gas-Phase and in Solution. J. Chem. Theory Comput. 2013, 9, 2209–2220. 10.1021/ct400021c. 26583715

[ref19] Brémond E.; Savarese M.; Adamo C.; Jacquemin D. Accuracy of TD-DFT Geometries: A Fresh Look. J. Chem. Theory Comput. 2018, 14, 3715–3727. 10.1021/acs.jctc.8b00311. 29883546

[ref20] Budzák S.; Scalmani G.; Jacquemin D. Accurate Excited-State Geometries: A CASPT2 and Coupled-Cluster Reference Database for Small Molecules. J. Chem. Theory Comput. 2017, 13, 6237–6252. 10.1021/acs.jctc.7b00921. 29140697 PMC5729545

[ref21] Huet T. R.; Godefroid M.; Herman M. The Ã Electronic State of Acetylene - Geometry and Axis-Switching Effects. J. Mol. Spectrosc. 1990, 144, 32–44. 10.1016/0022-2852(90)90306-b.

[ref22] Clouthier D. J.; Ramsay D. A. The Spectroscopy of Formaldehyde and Thioformaldehyde. Annu. Rev. Phys. Chem. 1983, 34, 31–58. 10.1146/annurev.pc.34.100183.000335.

[ref23] Ernsting N. P.; Pfab J.; Römelt J. Geometry Changes Accompanying Electronic Excitation of Nitrosomethane in the 650 nm Region. J. Chem. Soc., Faraday Trans. 2 1978, 74, 2286–2294. 10.1039/f29787402286.

[ref24] Jagau T. C.; Gauss J. Ground and excited state geometries via Mukherjee’s multireference coupled-cluster method. Chem. Phys. 2012, 401, 73–87. 10.1016/j.chemphys.2011.10.016.

[ref25] Köse M. E. How to Predict Excited State Geometry by Using Empirical Parameters Obtained from Franck-Condon Analysis of Optical Spectrum. ChemPhysChem 2021, 22, 2078–2092. 10.1002/cphc.202100437. 34351030

[ref26] Frisch M. J.; Trucks G. W.; Schlegel H. B.; Scuseria G. E.; Robb M. A.; Cheeseman J. R.; Scalmani G.; Barone V.; Petersson G. A.; Nakatsuji H.; Li X.; Caricato M.; Marenich A. V.; Bloino J.; Janesko B. G.; Gomperts R.; Mennucci B.; Hratchian H. P.; Ortiz J. V.; Izmaylov A. F.; Sonnenberg J. L.; Williams; Ding F.; Lipparini F.; Egidi F.; Goings J.; Peng B.; Petrone A.; Henderson T.; Ranasinghe D.; Zakrzewski V. G.; Gao J.; Rega N.; Zheng G.; Liang W.; Hada M.; Ehara M.; Toyota K.; Fukuda R.; Hasegawa J.; Ishida M.; Nakajima T.; Honda Y.; Kitao O.; Nakai H.; Vreven T.; Throssell K.; Montgomery J. A.; Peralta J. E.; Ogliaro F.; Bearpark M. J.; Heyd J. J.; Brothers E. N.; Kudin K. N.; Staroverov V. N.; Keith T. A.; Kobayashi R.; Normand J.; Raghavachari K.; Rendell A. P.; Burant J. C.; Iyengar S. S.; Tomasi J.; Cossi M.; Millam J. M.; Klene M.; Adamo C.; Cammi R.; Ochterski J. W.; Martin R. L.; Morokuma K.; Farkas O.; Foresman J. B.; Fox D. J. Gaussian 16, Revision C.01; Gaussian, Inc.: Wallingford, CT, 2016.

[ref27] Kashinski D. O.; Chase G. M.; Nelson R. G.; Di Nallo O. E.; Scales A. N.; VanderLey D. L.; Byrd E. F. C. Harmonic Vibrational Frequencies: Approximate Global Scaling Factors for TPSS, M06, and M11 Functional Families Using Several Common Basis Sets. J. Phys. Chem. A 2017, 121, 2265–2273. 10.1021/acs.jpca.6b12147. 28182415

[ref28] Gierschner J.; Mack H. G.; Lüer L.; Oelkrug D. Fluorescence and absorption spectra of oligophenylenevinylenes: Vibronic coupling, band shapes, and solvatochromism. J. Chem. Phys. 2002, 116, 8596–8609. 10.1063/1.1469612.

[ref29] Köse M. E.; Schanze K. S. Prediction of Internal Reorganization Energy in Photoinduced Electron Transfer Processes of Molecular Dyads. J. Phys. Chem. A 2020, 124, 9478–9486. 10.1021/acs.jpca.0c09533. 33141580

[ref30] Sando G. M.; Spears K. G. Ab initio computation of the Duschinsky mixing of vibrations and nonlinear effects. J. Phys. Chem. A 2001, 105, 5326–5333. 10.1021/jp004230b.

[ref31] Köse M. E. An activated scheme for resonance energy transfer in conjugated materials. J. Chem. Phys. 2011, 135, 244512 10.1063/1.3673962. 22225174

[ref32] Christiansen O.; Stanton J. F.; Gauss J. A coupled cluster study of the 1(1)A(1g) and 1(1)B(2u) states of benzene. J. Chem. Phys. 1998, 108, 3987–4001. 10.1063/1.475801.

[ref33] Butler P.; Moss D. B.; Yin H.; Schmidt T. W.; Kable S. H. Spectroscopy of the A(B-1(2))-X((1)A(1)) transition of jet-cooled fluorobenzene: Laser-induced fluorescence, dispersed fluorescence, and pathological Fermi resonances. J. Chem. Phys. 2007, 127, 094303 10.1063/1.2759931. 17824735

[ref34] Miura M.; Aoki Y.; Champagne B. Assessment of time-dependent density functional schemes for computing the oscillator strengths of benzene, phenol, aniline, and fluorobenzene. J. Chem. Phys. 2007, 127, 084103 10.1063/1.2761886. 17764225

[ref35] Cvitaš T.; Hollas J. M.; Kirby G. H. Interpretation of Rotational Constants of First Singlet Excited State of Substituted Benzenes in Terms of Molecular Geometry. Mol. Phys. 1970, 19, 305–316. 10.1080/00268977000101311.

[ref36] Lombardi J. R.; Wallenstein R.; Hänsch T. W.; Friedrich D. M. High-Resolution 2-Photon Spectroscopy in 1B2u State of Benzene. J. Chem. Phys. 1976, 65, 2357–2366. 10.1063/1.433349.

[ref37] Gardner A. M.; Wright T. G. Consistent assignment of the vibrations of monosubstituted benzenes. J. Chem. Phys. 2011, 135, 114305 10.1063/1.3638266. 21950860

[ref38] Bernhardsson A.; Forsberg N.; Malmqvist P. A.; Roos B. O.; Serrano-Andrés L. A theoretical study of the B-1(2u) and B-1(1u) vibronic bands in benzene. J. Chem. Phys. 2000, 112, 2798–2809. 10.1063/1.480854.

[ref39] Pugliesi I.; Tonge N. M.; Cockett M. C. R. An excited state ab initio and multidimensional Franck-Condon analysis of the A B-1(2)<- X (1)A(1) band system of fluorobenzene. J. Chem. Phys. 2008, 129, 104303 10.1063/1.2970092. 19044909

[ref40] Baba M.; Kowaka Y.; Nagashima U.; Ishimoto T.; Goto H.; Nakayama N. Geometrical structure of benzene and naphthalene: Ultrahigh-resolution laser spectroscopy and ab initio calculation. J. Chem. Phys. 2011, 135, 054305 10.1063/1.3622766. 21823698

[ref41] Li J.; Lin C. K.; Li X. Y.; Zhu C. Y.; Lin S. H. Symmetry forbidden vibronic spectra and internal conversion in benzene. Phys. Chem. Chem. Phys. 2010, 12, 14967–14976. 10.1039/c0cp00120a. 20949142

[ref42] Kisiel Z.; Białkowska-Jaworska E.; Pszczółkowski L. The millimeter-wave rotational spectrum of fluorobenzene. J. Mol. Spectrosc. 2005, 232, 47–54. 10.1016/j.jms.2005.02.006.

[ref43] Lipp E. D.; Seliskar C. J. Vibrational-Spectrum of Fluorobenzene. J. Mol. Spectrosc. 1978, 73, 290–304. 10.1016/0022-2852(78)90221-7.

[ref44] Callomon J. H.; Dunn T. M.; Mills I. M. Rotational Analysis of 2600A Absorption System of Benzene. Philos. Trans. R. Soc., A 1966, 259, 499–532.

[ref45] Fally S.; Carleer M.; Vandaele A. C. UV Fourier transform absorption cross sections of benzene, toluene, meta-, ortho-, and para-xylene. J. Quant. Spectrosc. Radiat. Transfer 2009, 110, 766–782. 10.1016/j.jqsrt.2008.11.014.

[ref46] Keller-Rudek H.; Moortgat G. K.; Sander R.; Sörensen R. The MPI-Mainz UV/VIS Spectral Atlas of Gaseous Molecules of Atmospheric Interest. Earth Syst. Sci. Data 2013, 5, 365–373. 10.5194/essd-5-365-2013.

[ref47] He R. X.; Yang L.; Zhu C. Y.; Yamaki M.; Lee Y. P.; Lin S. H. Franck-Condon simulation of the A B-1(2) -> X (1)A(1) dispersed fluorescence spectrum of fluorobenzene and its rate of the internal conversion. J. Chem. Phys. 2011, 134, 094313 10.1063/1.3559454. 21384975

[ref48] Palmer M. H.; Ridley T.; Vrønning Hoffmann S. V.; Jones N. C.; Coreno M.; de Simone M.; Grazioli C.; Zhang T.; Biczysko M.; Baiardi A.; Peterson K. A. Combined theoretical and experimental study of the valence, Rydberg and ionic states of fluorobenzene. J. Chem. Phys. 2016, 144, 204305 10.1063/1.4949548. 27250304

